# Effects of Adenosine Extract from *Pholiota adiposa* (Fr.) Quel on mRNA Expressions of Superoxide Dismutase and Immunomodulatory Cytokines

**DOI:** 10.3390/molecules18021775

**Published:** 2013-01-29

**Authors:** Chang Rong Wang, Wen Tao Qiao, Ye Ni Zhang, Fang Liu

**Affiliations:** Department of Microbiology, College of Life Science, Nankai University, Tianjin 300071, China

**Keywords:** adenosine, *Pholiota adiposa*, SOD, immunomodulatory cytokines

## Abstract

*Pholiota adiposa* is a kind of edible mushroom which has long been known for its health care applications. To reveal the exact mechanism of its protective functions in humans, in this study we isolated and identified the active compound PB3 of *P. adiposa* for the first time by a combination of chromatography techniques, including NKA macroporous resin and Sephadex G-15. PB3, with molecular mass of 267.2 Da and molecular formula of C_10_H_13_N_5_O_4_ discovered by mass spectrum (MS) was identified to be adenosine. Mice were injected intraperitoneally with purified fraction PB3. Seven days after injection, we found a 1.5-fold increase of IL10 at the mRNA level, while a down regulated expression of IL-2, IL-6 and IFN-γ to 49.0%, 56.9% and 73.4%, respectively, was detected in spleen by real-time quantitative PCR. What’s more, SOD expression level was significantly increased by 1.6-fold compared to control. Fraction PB3 displayed anti-inflammatory potency and heightened SOD activity on the transcriptional level, which could be considered of further pharmaceutical or medication value.

## 1. Introduction

*Pholiota adiposa* is one of the edible mushrooms grows wildly in China, which have been the subject of considerable attention for their health-care applications. It is rich in essential amino acids, vitamins, trace elements and proteins, including bio-active enzymes [1]. The extracts from fruiting bodies of *P. adiposa* have been reported to have a variety of functions such as antioxidative [2], antitumor [3] and antimicrobial properties [4]. 

Adenosine, which was widely known for its cardioprotective effects [5], is the active ingredient of many medicinal mushrooms, including *Cordyceps sinensis* and *Ganoderma lucidum*. It binds to specific receptors which are coupled to G-proteins and additionally to various effector-systems. Current studies have shown that endogenous and exogenous adenosine is involved in myocardial ischemia protection which protects the heart from the deleterious effects of inadequate blood flow and oxygen supply [5]. Adenosine provides protective effects against lung ischemia-reperfusion injuries by inhibiting the production of proinflammatory cytokines IL-6 and increasing inhibitory cytokine IL-10 [6]. Moreover, adenosine activates an adenosine receptor coupled to a pertussis toxin-sensitive G protern and phospholipase C (PLC), leading to the production of diacylglycerol (DAG) and inositol 1,4,5-trisphosphate thus increasing intracellular Ca^2+^ mobilization from the endoplasmic reticulum while DAG activates protein kinase C together with Ca^2+^. It is proposed that protein kinase C directly phosphorylates and activates antioxidant enzymes or phosphorylates an intermediate substrate which promotes activation of antioxidant enzymes including SOD and catalase (CAT) [7].

Cytokines are critical regulatory factors and play important roles in the immune system. Interleukin 6 (IL-6) is secreted by T cells and macrophages to stimulate immune response. IL-6 has been shown to interact with interleukin-6 receptors expressed on the cell surface and intracellular compartments and induces intracellular signaling cascades that give rise to inflammatory cytokine production [8]. Interleukin 10 (IL-10), known as human cytokine synthesis inhibitory factor (CSIF), is an anti-inflammatory cytokine released by cytotoxic T-cells. IL-10 is a cytokine with pleiotropic effects in immunoregulation and inflammation for counteracting the hyperactive immune response in the human body [9]. IL-10 is capable of inhibiting synthesis of pro-inflammatory cytokines including Interleukin 2 (IL-2) and interferon-γ (IFN-γ) which are made by cells such as macrophages and regulatory T-cells.

The aim of this study was to isolate active adenosine fraction from *P. adiposa*. Its effects on mRNA expression of superoxide dismutase and immunoregulator factors induced by LPS injection were studied to explore its regulation function for effects of adenosine on the transcriptional level were barely reported.

## 2. Results and Discussion

### 2.1. Chromatographic Action of Extract from P. adiposa

**Figure 1:**
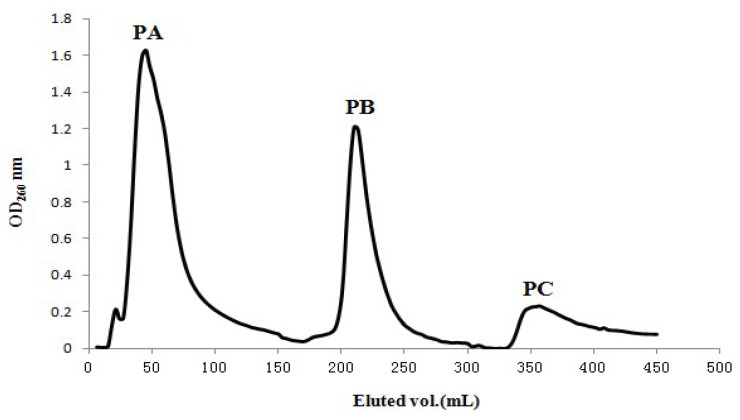
Elution profile of crude extract P from NKA macroporous resin column.

**Figure 2:**
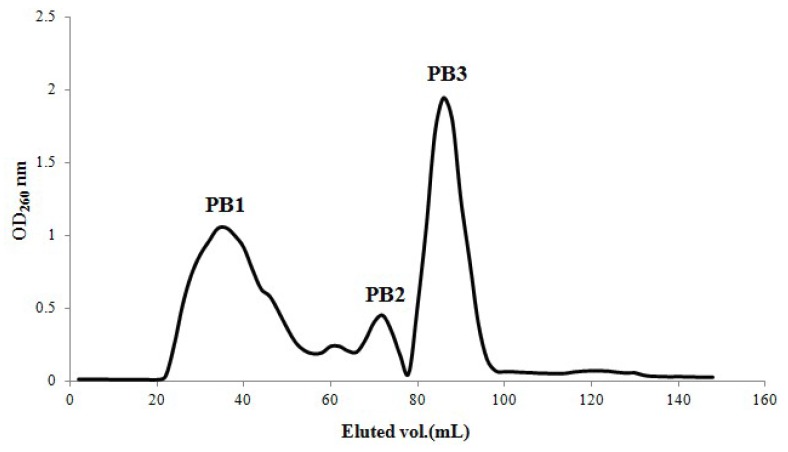
Elution profile of PB from Sephadex G15.

The elution profile of fractions PA, PB and PC from crude extract P on NKA macroporous resin were shown in Figure 1. Then fraction PB was chosen for analysis on Sephadex G15 (Figure 2). The resulting fractions PB1, PB2 and PB3 were collected separately.

Fractions were collected through sample elution with 150 mL distilled water, correspond to the marks on the x axis between 0 and 150 mL; fractions collected through sample elution with 150 mL of 30% ethanol, correspond to the marks on the x axis between 150 and 300 mL; and fractions collected through sample elution with 150 mL of 70% ethanol, correspond to the marks on the x axis between 300 and 450 mL.

Two kinds of chromatography were thus used in this study to isolate adenosine from *P. adiposa* in an efficient way. Macroporous resin polymers contain a permanent network of pores independent of the swelling state of the resin. The nonpolar resin NKA with its highly cross-linked structure is a good carrier used in low aqueous media which was appropriate for adenosine with its weak polarity. Sephadex is a gel filtration medium prepared by crosslinking dextran with epichlorohydrin. Different types of Sephadex differ in their degree of cross-linking and hence in their degree of swelling and their molecular fractionation range. Sephadex G-15 is a well established gel filtration medium for removing contaminants from small biomolecules, including adenosine, in a single step. Moreover, we purified the sample using only distilled water and methanol which were safer than other organic solvents such as chloroform, ethyl acetate and ether.

### 2.2. Identification of the Extract PB3

**Figure 3:**
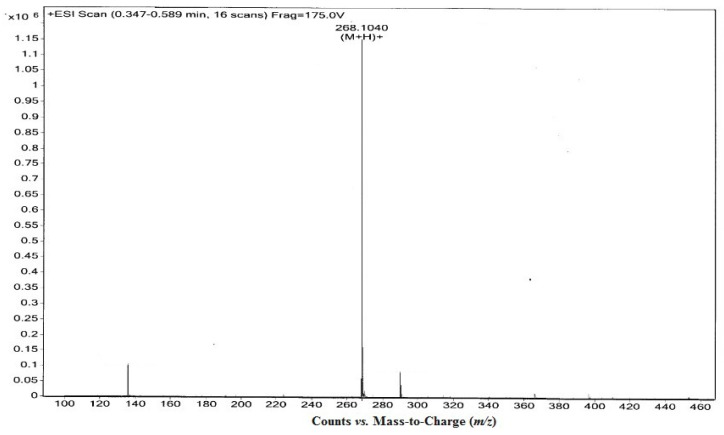
Mass spectrometric detection of chromatographic fraction PB3 of *P. adiposa* fruiting bodies.

**Figure 4:**
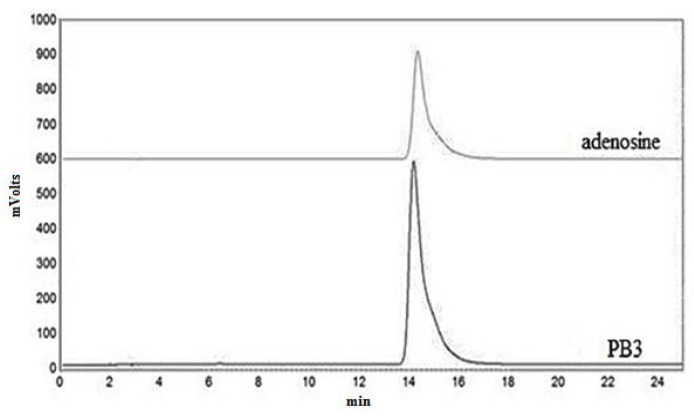
Chromatogram of fraction PB3 of *P. adiposa* and standard adenosine in the same mobile phase.

Fraction PB3 was purified on a HPLC preparative column followed by an analytical column eluting with 15% methanol. Concurrently, the retention time of PB3 and standard adenosine matched well (Figure 3). Moreover, mass spectrometric analyses using electrospray ionization mass spectrometry (ESI-MS) showed that the *m/z* [M+H]^+^ of PB3 was 268.1040 Da, which corresponded to a molecular mass of 267.2 Da (Figure 4) and a molecular formula of C_10_H_13_N_5_O_4_. This matched the data in the online database for adenosine [10]. Therefore, it could be concluded that PB3 was adenosine.

### 2.3. Effect of PB3 on mRNA Expression of SOD 

Adenosine exerts a range of generally beneficial effects in the heart and vessels [11]. It has been reported to be isolated from precious medicinal mushrooms, including *C. sinensis* and *G. lucidum*, as a significant active ingredient. In this study, adenosine was extracted for the first time from the edible mushroom *P. adiposa*. This suggested that *P. adiposa* might have potential for medicinal purposes. 

Adenosine is an important regulator within the cardiovascular system where it interacts with four adenosine receptor subtypes on constituent cardiac and vascular cells. These G-protein-coupled receptors mediate varied responses, from modulation of coronary flow, heart rate and contraction, to cardioprotection and inflammatory regulation [12]. Furthermore, it directly phosphorylates and activates antioxidant enzymes or phospholylates an intermediate substrate which promotes activation of antioxidant enzymes [7]. The effects of adenosine on the transcriptional level however were barely reported. To fill this gap, the mRNA expressions of superoxide dismutase and immunomodulatory cytokines were tested in this study. The SOD gene expression level treated with fraction PB3 was significantly increased by 1.6-fold compared to the control (Table 1). This showed that PB3 could enhance the expression of the antioxidant enzyme SOD genes.

The damaging effects of oxidative stress are related to the inability of cellular defense enzymes to adequately scavenge ROS and free radicals. Antioxidant enzymes play an important role in protecting cells from oxidative stress induced by inflammatory response. SOD are enzymes that catalyze the dismutation of superoxide into oxygen and hydrogen peroxide. They are important antioxidant defenses in nearly all cells exposed to oxygen. In this study, it was shown that PB3 could enhance the mRNA expression of SOD to balance the internal redox environment on the transcriptional level. It also helped to relieve inflammatory damage. 

### 2.4. Effect of PB3 on mRNA Expression of Immunomodulatory Cytokines

Treatment with fraction PB3 brought about a 1.5-fold increase in the level of IL-10, while it down-regulated the expression of IL-2, IL-6 and IFN-γ to 49.0%, 56.9% and 73.4%, respectively (Table 1). The biological activity of adenosine isolated from different mushrooms was similar for its cardioprotective effects and immunoregulation functions, but there are few reports about adenosine extracted from edible mushrooms. This was the first chromatographic isolation of adenosine from the edible mushroom *P. adiposa*, which suggested that *P. adiposa* might have novel medicinal value. It was known that adenosine plays a biological role through cell surface adenosine receptors which have been a hot area of research for several years, but the functions of adenosine on the transcriptional level were barely reported. To fill this gap, mRNA expressions of immunomodulatory cytokines were tested *in vivo* by mice experiments which simulated the physiological state much better than cell lines.

Fraction PB3 showed anti-inflammatory effects by up-regulating the expression of cytokine synthesis inhibitory factor IL-10, while down-regulating the expression of proinflammatory cytokines, including IL-2, IL-6 and IFN-γ. IL-10 is classified as a class-2 cytokine which down-regulates the expression of Th1 cytokines to suppress inflammation [13]. It displays potent ability to inhibit the synthesis of pro-inflammatory cytokines such as IL-2 and IFN-γ made by type 1 T helper cells as the results in the study show. IL-10, which was up-regulated by PB3 in this study, has significant roles in regulation of the immune system to modulate inflammatory responses. IL-6 secreted by T cells and macrophages act as a pro-inflammatory agent to stimulate immune response during infection and after trauma. IL-6 signals through type I cytokine receptor complex which are present on the cell surface and intracellular compartments and induces intracellular signaling cascades that give rise to inflammatory cytokine production [14]. In this study, it was shown that PB3 could suppress the mRNA expression of IL-6 while it enhanced the expression of IL-10 which inhibits the synthesis of pro-inflammatory cytokines IL-2 and IFN-γ. In a word, PB3 played a role in anti-inflammatory effects on the transcriptional level to balance the immune system.

## 3. Experimental 

### 3.1. Reagents

The macroporous adsorption resin NKA was purchased from the Chemical Plant of Nankai University (Tianjin, China). Sephadex G15 was purchased from Hui De Yi Co., Ltd. (Beijing, China). Trizol and RT-PCR kits were purchased from Dingguo Biotechnology Development Center (Beijing, China). 2×SYBR Green Realtime PCR Master Mix was from TOYOBO Co., Ltd. (Tokyo, Japan). All other chemicals were of the highest quality available.

### 3.2. Animals

Male BALB/c mice, 5–6 weeks of age and with a body weight of 20 ± 2 g, were purchased from a local registered animal supplier. The mice were housed under normal laboratory conditions (25 ± 2 °C, 12 h : 12 h light-dark cycle) with free access to standard rodent chow and water.

### 3.3. Sample Preparation and Isolation of Adenosine

Dried *P. adiposa* cultured and collected in Henan Luoyang on September 2010, was provided by Mr. Jun Hou from Henan University of Science and Technology and then authenticated. The dried fruit bodies of *P. adiposa* were powdered and extracted with 70% ethanol (100 g : 2 L) under reflux. After filtration to remove debris, the filtrate (crude extract named P) was concentrated on a rotary evaporator.

The crude extract P was purified by chromatography with macroporous resins NKA, a kind of apolar cross-linked polystyrene, which has a bead size of 0.3–1.0 mm, a surface area of 570–590 m^2^/g, and an average pore diameter of 200–220 Å. P was dissolved in distilled water (500 mg/mL) and then chromatographed on a column of macroporous resin (2.5 × 35 cm). The inactive fraction named PA was eluted with distilled water. The resin was then eluted with 30% (v/v) ethanol, and the resulting fraction was named PB. Finally, the resin was regenerated with 70% (v/v) ethanol to get the fraction named PC. PB was chromatographed on a column of Sephadex G15 (1.2 × 60 cm) which was eluted with distilled water. The fractions collected were named PB1, PB2 and PB3. The fraction PB3 was purified with a Lab Alliance high-performance liquid chromatography (HPLC) system, including two Series III pumps, a 500 absorbance detector set at 260 nm, and connected to a Cs420x Hardware integrator (Lab Alliance, Tianjin, China). The columns used were a Merck (Darmstadt, Germany) reverse-phase C18 ODS preparative (250 × 10 mm) and analytical column (250 × 4.6 mm). The eluent was 15% methanol under 30 °C at a flow rate of 3 mL/min for the preparative column and 1 mL/min for the analytical column.

### 3.4. Real-Time PCR

Ten mice were divided randomly into two groups. Control mice (group A) were treated with 0.9% (w/w) NaCl by intraperitoneal injection. PB3 mice (group B) were treated with PB3 (50 mg/kg). All mice were injected once a day for seven days. On the eight day, all mice received an intraperitoneal injection of purified LPS (derived from *E. coli* O55:B5; Sigma, St. Louis, MO, USA) at a dose of 2 mg/kg. All mice were sacrificed 12 h later and total splenic RNA were isolated by using the Trizol kit. 

The reaction mixture was composed of 1–5 μg total RNA, 0.5 mM dNTPs, 50 ng oligo (dT) primer, reverse transcription buffer, and 3 units of reverse transcriptase. It was heated at 65 °C for 5 min, then conducted at 42 °C for 30 min, and finally inactivated by heating to 95 °C for 5 min.

Real-time PCR reaction mixture contained upstream primer (1 μL, 500 pmol), downstream primer (1 μL, 500 pmol), cDNA (1 μL), ddH_2_O (7 μL) and 2×SYBR Green Realtime PCR Master Mix (10 μL). The following thermocycler program was used for real-time PCR: 1 min preincubation at 95 °C, followed by 40 cycles of incubation at 94 °C for 15 s, 55 °C for 30 s, 72 °C for 45 s. The primers of super oxygen dehydrogenises (SOD), IL-2, IL-6, IL-10, interferon-γ (INF-γ) and glyceraldehyde 3-phosphate dehydrogenase (GAPDH) are shown in Table 2 [15]. The 2^−ΔΔCt^ method [16] was used to analyze the results. GAPDH was the internal control.

## 4. Conclusions

In this study, adenosine was isolated for the first time from the mushroom *P. adiposa*. The fraction PB3 which was identified to be adenosine displayed potent anti-inflammatory effects by enhancing the expression of anti-inflammatory cytokines IL-10, as well as suppressing the expression of inflammatory factors IL-6, IL-2 and IFN-γ. Fraction PB3 helped balance the redox system and immune system on the transcriptional level. This suggests that adenosine might not only have cardioprotective effects, but also potential in the treatment of inflammation.

## Figures and Tables

**Table 1:** Effects of PB3 on the expression of target genes (n = 5).

Gene	ΔCt *	ΔΔCt **	2^−ΔΔCt^
IL-2	9.43 ± 0.15	1.03	0.490
IL-6	7.60 ± 0.19	0.81	0.569
IL-10	8.05 ± 0.05	−0.57	1.481
IFN-γ	7.81 ± 0.17	0.45	0.734
SOD	3.95 ± 0.17	−0.70	1.621

The ΔCt values are mean ± S.D. (n = 5); * ΔCt = Ct_gene_ − Ct_GAPDH_. ** ΔΔCt = ΔCt_sample_ − ΔCt_control_.

**Table 2:** Primers for Real-time PCR.

Gene	Sequence		Product
SOD	upstream:	5'-GCAGGGAACCATCCACTT-3'	215 bp
	downstream:	5'-TGCCCAGGTCTCCAACAT-3'	
IL-2	upstream:	5'-TGAACTTGGACCTCTGCG-3'	220 bp
	downstream:	5'-AGGGCTTGTTGAGATGATGC-3'	
IL-6	upstream:	5'-TTCTTGGGACTGATGCTG-3'	380 bp
	downstream:	5'-CTGGCTTTGTCTTTCTTGTT-3'	
IL-10	upstream:	5'-ACCAAAGCCACAAAGCAG-3'	249 bp
	downstream:	5'-GGAGTCGGTTAGCAGTATG-3'	
IFN-r	upstream:	5'-TGAGACAATGAACGCTAC-3'	142 bp
	downstream:	5'-TTCCACATCTATGCCACT-3'	
GAPDH	upstream:	5'-TCAACGGCACAGTCAAGG-3'	470 bp
	downstream:	5'-ACCAGTGGATGCAGGGAT-3'	
